# Assessment of early treatment response on MRI in multiple myeloma: Comparative study of whole-body diffusion-weighted and lumbar spinal MRI

**DOI:** 10.1371/journal.pone.0229607

**Published:** 2020-02-27

**Authors:** Miyuki Takasu, Shota Kondo, Yuji Akiyama, Yuji Takahashi, Shogo Maeda, Yasutaka Baba, Takakazu Kawase, Tatsuo Ichinohe, Kazuo Awai

**Affiliations:** 1 Department of Diagnostic Radiology, Graduate School of Biomedical Sciences, Hiroshima University, Hiroshima, Japan; 2 Department of Hematology and Oncology, Research Institute for Radiation Biology and Medicine, Hiroshima University, Hiroshima, Japan; George Washington University, UNITED STATES

## Abstract

**Objectives:**

To compare remission status at completion of chemotherapy for multiple myeloma (MM) with changes in total diffusion volume (tDV) calculated from whole-body diffusion-weighted imaging (WB-DWI) and fat fraction (FF) of lumbar bone marrow (BM) by modified Dixon Quant (mDixon Quant) soon after induction of chemotherapy, and to assess the predictive value of MRI.

**Methods:**

Fifty patients (mean age, 66.9 ± 10.5 years) with symptomatic myeloma were examined before and after two cycles of chemotherapy. From WB-DWI data, tDV was obtained with the threshold for positive BM involvement. Mean FF was calculated from lumbar BM using the mDixon Quant sequence. At the completion of chemotherapy, patients were categorized into a CR/very good PR (VGPR) group (n = 15; mean age, 67.6 ± 10.3 years) and a PR, SD or PD group (n = 35; mean age, 69.1 ± 8.6 years). ROC curves were plotted to assess performance in predicting achievement of CR/VGPR.

**Results:**

At second examination, serum M protein, β_2_-microglobulin, and tDV were significantly decreased and hemoglobin, mean ADC, and FF were significantly increased in the CR/VGPR group and serum M protein was significantly increased in the PR/SD/PD group. The general linear model demonstrated that percentage changes in FF and M protein contributed significantly to achieving CR/VGPR (P = 0.02, P = 0.04, respectively). AUCs of ROC curves were 0.964 for FF and 0.847 for M protein.

**Conclusions:**

Early change in FF of lumbar BM and serum M protein soon after induction of chemotherapy contributed significantly to prediction of CR/VGPR.

## Introduction

Multiple myeloma (MM) is a B-cell malignancy characterized by accumulation of malignant plasma cells that secrete monoclonal protein (M protein). The introduction of several new drugs has led to improved survival for MM patients [1, 2]. However, treatment response to chemotherapy for MM is highly heterogeneous, due to differences in protein manifestations and genetic alterations [3]. Accurate assessment of treatment response is therefore necessary.

Response evaluation in MM has been based on the assessment of serum M protein and serum free light chain (sFLC) values as surrogates for tumor burden as well as bone marrow (BM) plasma-cell quantitation from BM marrow aspirates [4]. Modern imaging modalities including ^18^F-fluorodeoxyglucose positron emission tomography–computed tomography (FDG-PET/CT) and MRI have recently been reported to be useful in evaluating the depth of treatment response [5, 6]. The International Myeloma Working Group (IMWG) therefore incorporated these imaging modalities into new response categories in 2016 to allow uniform reporting within and outside of clinical trials [4].

Whole-body MRI provides a measure to repeatedly assess the extent of disease in the entire BM during the course of the disease, and may be useful for predicting disease outcome from diagnosis [7, 8]. With whole-body diffusion-weighted imaging (WB-DWI) for myeloma, quantitative assessment of the tumor cell burden and response to chemotherapy has become available, using the apparent diffusion coefficient (ADC) to quantify disease [9, 10]. The relatively long acquisition time is the main drawback of whole-body MRI in clinical practice, especially for patients MM who often experience significant skeletal pain.

Traditionally, visual assessment has commonly been used to differentiate infiltrative pathology from normal hematopoietic BM. Hematopoietic red marrow usually has fatty yellow marrow intermixed and most lesions interfere with this medullary water-fat balance [11]. Changes in the BM associated with MM, such as replacement of the BM fat by myeloma cell infiltrations or elevated numbers of hematopoietic cells, reduce the abundance of fat and increase water content [12, 13]. The evaluation of vertebral BM fat content based on the water-fat chemical shift difference has gained significant attention [14, 15].

Chemical shift imaging was originally described in 1984 as a 2-point Dixon method that can provide both in-phase and opposed-phase images [16]. Since the mid-2000s, multi-echo Dixon water and fat separation has been used with arbitrary echo times to allow for more flexible sequence designs. One method, iterative decomposition with echo asymmetry and least-squares (IDEAL) [17] produces excellent discrimination between water and fat. As a result, a quantitative measure of fat content can be performed using the IDEAL technique. For lumbar BM of MM, the fat signal fraction of lumbar BM without a focal lesion was demonstrated to have potential for discriminating between symptomatic and asymptomatic myeloma [18, 19]. With advances in the Dixon technique, a 2-point Dixon method was developed as a modified Dixon (mDixon) with flexible echo times for water-fat separation, utilizing the referenced seven-peak spectral model [20]. By calculating the two shortest echo times, the mDixon technique provided an improved signal-to-noise ratio (SNR) while maintaining high spatial resolution.

Recent studies in 2017 and 2018 using an mDixon Quant sequence with six echoes, seven fat peaks and T2* correction in a short acquisition time was found to enable robust water-fat separation and to offer high-quality fat quantification [21–23].

The Myeloma Response Assessment and Diagnosis System imaging was recently proposed to promote standardization of whole-body MRI and response assessment, mainly based on ADC changes in myeloma [24]. The purpose of this study was to compare remission status at completion of chemotherapy with changes in MRI biomarkers obtained using advanced MRI techniques, including total diffusion volume (tDV) calculated from WB-DWI and fat fraction (FF) of lumbar BM by mDixon Quant soon after induction of chemotherapy, and to assess the predictive value of MRI.

## Materials and methods

### Study cohort

This retrospective, single-institution study was approved by the Institutional Review Board of Hiroshima University Hospital, with a waiver for informed consent.

We searched a computerized database and reviewed the medical records of all patients between July 2016 and November 2018. The criteria used for diagnosis were taken from the criteria of the IMWG [7]. Patients were included if they underwent whole-body MRI including both WB-DWI and mDixon Quant sequence of lumbar BM before starting chemotherapy and after two cycles of the chemotherapy. Patients with an interval of > 10 weeks between the initial and second MRI were excluded.

Twenty-three men (mean age, 61.2 years; range, 43–81 years) and 27 women (mean age, 65.7 years; range, 55–87 years) were included in the study. All patients had symptomatic myeloma. The M protein comprised immunoglobulin (Ig)G (28 patients), IgA (nine patients), IgD (three patients), or Bence Jones protein (10 patients). No patients had non-secretory myeloma. Using the Durie-Salmon Staging System, 30 patients were classified as stage ⅢA, five patients as stage ⅢB, 13 patients as stage ⅡA, and two patients as stage ⅡB. Using the revised International Staging System, 15 patients were classified as stage Ⅲ, 34 patients as stage II, and one patient as stage Ⅰ. Chromosome 17p deletion was present in one patient, translocation t(14;16) was present in one patient, and translocation t(4;14) was present in one patient. No patients had non-secretory myeloma. Of these 50 patients, 26 had been newly diagnosed with MM and the remaining 24 patients had received up to 5 prior chemotherapy regimens (median, 2 regimens). We did not exclude the latter 24 patients with a history of prior chemotherapy because the aim of this study was to compare the performance of imaging sequences per patient. Of these 24 previously treated patients, 9 experienced relapsed myeloma, 7 had relapsed and refractory myeloma, and 8 had primary refractory myeloma according to the international uniform response criteria for MM by the IMWG [25].

### MRI

Whole-body MRI examinations were performed using a 3-T system (Ingenia; Philips Healthcare) with a maximum gradient amplitude of 40 mT/m and a maximum slew rate of 200 mT/m/s equipped with head, anterior torso array, and integrated posterior coils. Patients were imaged in the supine position with 4 stacks covering vertex to knees. An overlap of several centimeters was applied between each station.

Imaging parameters are summarized in Table 1 (http://dx.doi.org/10.17504/protocols.io.[dx.doi.org/10.17504/protocols.io.bavbie2n]). A whole-body coronal 3D-spoiled gradient-echo pulse sequence (mDixon Quant) was performed with six evenly spaced echoes (first echo time, 1.15 ms; echo spacing, 1.15 ms).

**Table 1:** MRI sequence protocol.

Parameter	Sequence						
	Whole-spine sagittal T1-weighted	Whole-spine sagittal STIR	Whole-body axial T2-weighted	Whole body coronal T1-weighted	Whole-body coronal DWIBS	Lumbar spinal DWI	Whole-body coronal mDixon Quant
Sequence type	FSE	STIR	FSE	FSE	STIR	EPI	3D SPGR
Time (ms)							
TR	404	5693	1000	515	5411	8000	5.7
TE	10	70	70	15	70	84	Six evenly spaced echoes
Inversion time	N/A	200	N/A	N/A	250	N/A	N/A
Slice thickness (mm)	4	4	6	5	4	4	6
Number of slices per station	15	15	40	34	50	11	64
In-plane pixel size (mm)	1.4×2.7	1.5×2.2	1.1×1.5	1.7×3.5	2.3×3.5	2.7×2.7	2.4×2.4
Bandwidth/pixel (Hz)	576	625	359	435	2535	2024	128
Acquisition time (min)	6	6	6	6	8	6.5	1.3
b Value (s/mm^2^)	N/A	N/A	N/A	N/A	0 and 1000	0, 40, 80, 140, 200, 500, 1000, 1500, and 2000	N/A

DWIBS, diffusion-weighted whole-body imaging with background body signal suppression; mDixon Quant, modified Dixon Quant; FSE, fast spin-echo; STIR, short tau inversion recovery; EPI, echo-planar imaging: 3D SPGR, 3D spoiled gradient- echo; N/A, not applicable.

### Image analysis

All MR images were reviewed by two authors with 25 years of experience in spinal imaging (M.T.) and musculoskeletal imaging (Y.B.), respectively. They evaluated the pattern of BM infiltration on T1-weighted images and STIR images (i.e., normal, focal-dominant, combined diffuse and focal pattern, or diffuse-dominant) in a blinded and independent manner. Disagreements were then discussed to reach consensus. The diffuse-dominant pattern was defined as a diffuse hypo- or iso-intense signal in spinal BM compared with nondegenerated intervertebral discs. If no focal lesion was present and signal intensity in the spinal BM was grossly homogeneous and brighter than nondegenerated intervertebral discs, a normal pattern was assigned. When one or more focal bone lesions greater than 5 mm in diameter was present, a focal-dominant pattern was assigned if signal intensity in spinal BM was grossly homogeneous and greater than nondegenerated intervertebral discs. If one or more focal bone lesions greater than 5 mm in diameter was present, a combined diffuse and focal pattern was assigned if signal intensity in spinal BM was grossly inhomogeneous and lower than or similar to nondegenerated intervertebral discs. Inter- and intraobserver agreements were assessed with linear kappa statistics (κ <0.2, poor; κ = 0.21–0.40, fair; κ = 0.41–0.60, moderate; κ = 0.61–0.80, good; κ = 0.81–0.90, very good; and κ >0.90, excellent).

For WB-DWI (from skull base to knees), image processing was performed by one author, M.T., with 4 years of experience in reading WB-DWI studies using newly developed medical imaging software (BD score; PixSpace, Japan). Semi-automatic segmentation of myelomatous lesion in each patient was performed using the following steps:

1. On a maximum intensity projection (MIP) image calculated from computed DWI (b value = 999 s/mm^2^), the contrast in signal between disease and background tissues was maximized on visual inspection. A single optimal value of 97 was then determined as a background threshold for all studies to provide an initial classification of disease from background.
2. After removal of the background, images were converted into binary images with two gray levels with a gray-level threshold value. White and black regions corresponded to disease and space other than disease, respectively. The threshold value was automatically determined by the Otsu discriminant analysis method [26], in which statistical discriminant analysis is utilized to a gray-level histogram of the image, and an adequate threshold value is determined objectively for each image.
3. All segmentation results were displayed on a MIP and multi-planar reformat viewer. High signal areas outside the skeletal system (e.g., brain, lymph node, intestine) were manually removed by the author. From the remaining high-signal areas, voxels showing an ADC ≤2.0 were extracted to eliminate the influence of T2 shine-through effect.

As an estimate of tDV, voxels with an ADC above the threshold which was determined in the preliminary experiment described in the next paragraph were extracted from the remaining high signal areas for each patient. Mean ADC, ADC histogram features (25th and 75th percentiles, skewness, and kurtosis) from all voxels in the tDV were also calculated. The tDV included extramedullary lesions for 3 newly diagnosed patients and 4 previously treated patients.

In a preliminary experiment, we compared three ADC thresholds for positive BM involvement, including ADC >0.55 ×10^−3^ mm^2^/s based on a previous report [10], which corresponded to the diagnosis of a diffuse MRI pattern on sagittal T1-weighted images of the thoracic and lumbosacral spine, >0.45 ×10^−3^ mm^2^/s, and >0.65 ×10^−3^ mm^2^/s. The latter two thresholds were arbitrarily selected using differences of 0.10 ×10^−3^ mm^2^/s either side of the reported threshold of >0.55 ×10^−3^ mm^2^/s. We performed this preliminary experiment to verify the validity of using the same threshold as in the previous report, because the MR unit we used in this study differed from that in the previous report [10], by examining whether the difference in thresholds of ADC would significantly affect prediction of disease severity (i.e., Stage II vs Stage III in the Durie-Salmon Staging System).

Single-shot lumbar spinal DWI with 9 b-values was performed to assess perfusion in the lumbar BM. Two parameters of pseudodiffusion coefficient and perfusion fraction were calculated using a biexponential model from mean signal intensity of the rectangular region of interest (ROI) within the BM of L1-L3 on mid-sagittal images, because these spinal levels were less affected by degenerative disc disease compared to lower lumbar elements, or were less likely to be fractured compared to lower thoracic elements BM regions with a focal lesion greater than 5 mm, degenerative disc disease, or fractures were carefully excluded from the ROIs. The ROIs for BM had an area of 245–480 mm^2^.

For whole-body coronal 3D mDixon Quant sequence, water-only, in-phase, opposed-phase, and fat-only images were obtained. Mean FF was calculated from the ratio of the signal intensity in the fat-only image divided by the signal intensity of the rectangular ROI on mid-coronal image of FF map from the same vertebrae used for a biexponential model. ROIs for BM had an area of 282–525 mm^2^.

Serological data, including serum M protein and kappa/lambda ratio were obtained.

### Statistical analysis

Treatment response was assessed according to the international uniform response criteria for MM by the IMWG [25]. Responses were defined as complete response (CR), very good partial response (VGPR), partial response (PR), stable disease (SD), or progressive disease (PD). For patients whose response was CR or VGPR, best response was defined as the best recorded level of response within the first 12 months. For patients with response of SD or PD, response assessments were performed before the next therapy was initiated.

Patients were categorized into the following two groups: patients who achieved CR or VGPR (CR/VGPR group; n = 15) and patients who showed PR, SD or PD (PR/SD/PD group; n = 35) at the completion of chemotherapy. We considered a responder as someone who achieved CR or VGPR because a recent meta-regression analysis of patients with MM found no association between response outcomes of CR and VGPR regarding survival [27]. Characteristics, biochemistry results, and MRI-derived indices of patients at baseline were compared with the Scheffe post-hoc test or Kruskal-Wallis test. The Cochrane-Armitage trend test was used to compare κ/λ ratio and the pattern of BM infiltration. Within-group changes of indices from baseline to second examination after 2 cycles of chemotherapy were compared using the Wilcoxon signed-rank test and expressed as percentage changes. Next, between-group differences in percentage changes were assessed with the Scheffe post-hoc test or Kruskal-Wallis test.

A multivariate general linear model was constructed to identify the best predictors of achievement of CR/VGPR. Variables showing values of *P* <0.05 on univariate analysis were included in the multivariate analysis.

Receiver operating characteristic (ROC) curves were performed to assess performance for predicting achievement of CR/VGPR.

Probability values were considered significant for values of *P* <0.05. All analyses were performed with a spreadsheet application (Office Excel version 3.00; Microsoft).

## Results

### Preliminary experiment

No significant difference was observed in areas under the ROC curve (AUCs) among the three groups of tDV calculated from each threshold for dividing patients with Stage Ⅱ disease from those with Stage Ⅲ using the χ^2^ test. AUCs were 0.654 for >0.45 ×10^−3^ mm^2^/s, 0.692 for >0.55 ×10^−3^ mm^2^/s, and 0.668 for >0.65 ×10^−3^ mm^2^/s. We therefore selected 0.55 ×10^−3^ mm^2^/s as a threshold to calculate tDV, based on the previous report [10].

### Patient characteristics

Of the 50 patients, 13 were treated with lenalidomide and dexamethasone, 13 were treated with bortezomib and dexamethasone, 8 were treated with pomalidomide and dexamethasone, 4 were treated with lenalidomide, bortezomib, and dexamethasone, 4 were treated with carfilzomib, lenalidomide, and dexamethasone, 3 were treated with daratumumab, lenalidomide, and dexamethasone, 3 were treated with bortezomib, cyclophosphamide, and dexamethasone, and 2 were treated with elotuzumab, lenalidomide, and dexamethasone, during the periods analyzed for the study.

Of the 50 patients, 11 (22.0%, including 3 patients with prior chemotherapy) were in CR, 4 (8.0%, no patients with prior chemotherapy) in VGPR, 3 (6.0%, including 1 patient with prior chemotherapy) in PR, 14 (28.0%, including 6 patients with prior chemotherapy) were in SD, and 18 (36.0%, including 14 patients with prior chemotherapy) were in PD.

### Performance of MRI-derived measurements

No significant differences in patient age (*P* = 0.90), biochemistry results (*P =* 0.30–0.82), or MRI-derived indices, including the pattern of BM infiltration, FF, and tDV (*P =* 0.14–0.90) at baseline examination, were evident between groups (Table 2). Interobserver agreement was moderate (κ = 0.62). Intraobserver agreement was moderate (κ = 0.62) for Observer 1 and very good (κ = 0.81) for Observer 2. At the first MRI examination, the patient population showed the following patterns of BM alteration on MRI: 6 patients (12.0%) had a focal-dominant pattern, 32 (64.0%) had a combined diffuse and focal pattern, and 12 (24.0%) had a diffuse pattern. No patients had a normal BM pattern. The combined diffuse and focal pattern was the dominant pattern in this cohort. This could be related to the fact that 20 of the 32 patients had received prior chemotherapy.

**Table 2:** Patient characteristics, biochemistry, and MRI-derived indices at baseline.

	CR/VGPR group	PR/SD/PD group	*P*
Number of patients	15	35	N/A
Age (years)	67.6 ± 10.3	69.1 ± 8.6	0.90
Laboratory data			
Serum M protein (mg/dl)	1546 ± 722	1987 ± 652	0.63
Albumin (g/dl)	3.72 ± 0.60	3.81 ± 0.67	0.76
Lactate dehydrogenase (U/l)	191 ± 42	213 ± 25	0.82
β_2_-microglobulin (mg/l)	4.50 ± 1.47	3.52 ± 2.02	0.41
Kappa/lambda ratio			0.30
0.125–8 (number of patients)	6	11	
<0.125 or >8 (number of patients)	9	24	
Hemoglobin (g/dl)	10.5 ± 2.2	11.5 ± 1.5	0.46
MRI data			
Pattern of BM infiltration			0.72
Normal	0	0	
Focal-dominant	3	3	
Combined diffuse and focal	8	24	
Diffuse-dominant	4	8	
Whole-body DWI			
Total diffusion volume (ml)	111.4 ± 75.2	64.3 ± 15.2	0.14
Mean ADC (×10^−3^ mm^2^/s)	1.076 ± 0.312	1.130 ± 0.242	0.45
Skewness	1.236 ± 1.107	1.464 ± 0.225	0.67
Kurtosis	6.543 ± 3.543	6.971 ± 1.462	0.83
25th percentile	0.784 ± 0.252	0.752 ± 0.268	0.87
75th percentile	1.131 ± 0.381	1.142 ± 0.508	0.85
Spinal DWI			
Perfusion fraction	0.247 ± 0.153	0.253 ± 0.187	0.90
D* (×10^−3^ mm^2^/s)	256.2 ± 322.5	286.3 ± 94.6	0.84
mDixon Quant			
Fat fraction (%)	32.2 ± 24.7	44.2 ± 22.4	0.14

Values represent mean ± standard deviation or median [range].

CR, complete response; VGPR, very good partial response; PR, partial response; SD, stable disease; PD, progressive disease; N/A, not applicable; BM, bone marrow; ADC, apparent diffusion coefficient; D*, pseudodiffusion coefficient.

**Fig 1:**
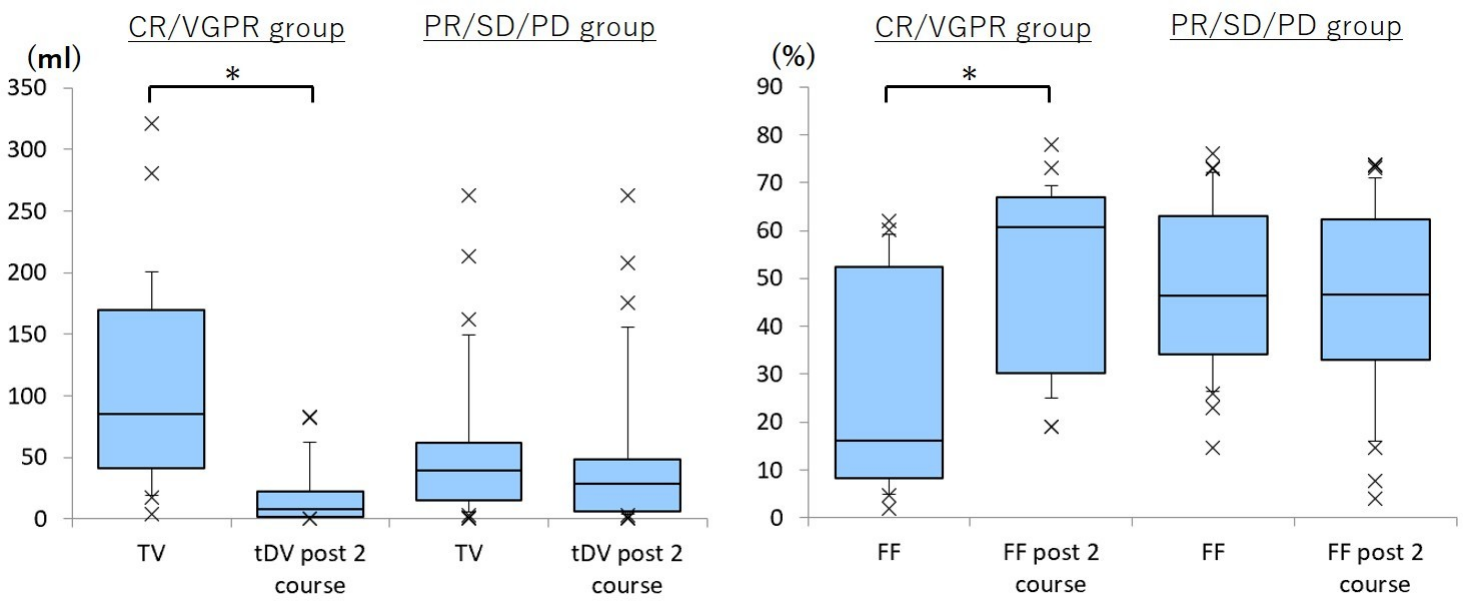
Boxplot of percentage changes in total diffusion volume (tDV) calculated from whole-body DWI and fat fraction (FF) of lumbar bone marrow by mDixon Quant during two courses of chemotherapy. At the second examination, tDV is significantly decreased and FF is significantly increased in the CR/VGPR group. The PR/SD/PD group includes patients showing responses to chemotherapy ranging from partial response to progressive disease. *, P < 0.05.

At the second examination, serum M protein, β_2_-microglobulin, and tDV were significantly decreased and hemoglobin, mean ADC, and FF were significantly increased in the CR/VGPR group, while serum M protein was significantly increased in the PR/SD/PD group (Table 3 and Fig 1).

**Table 3:** Percentage changes in serological and MRI-derived indices at second MRI.

	CR/VGPR group	PR/SD/PD group	*P*
Laboratory data			
Serum M protein	-46.2 ± 40.6*	93.2 ± 180.7*	0.02^+^
Albumin	8.35 ± 17.6	-1.08 ± 3.3	0.12
Lactate dehydrogenase	-0.32 ± 21.3	9.8 ± 7.9	0.24
β2-microglobulin	-24.6 ± 32.3*	13.0 ± 36.1	0.02^+^
Kappa/lambda ratio			
Normalized (number of patients)	7	10	
Non-normalized (number of patients)	8	25	0.25
Hemoglobin	12.0 ± 8.4*	-0.61 ± 17.8	0.02^+^
MRI data			
Whole-body DWI			
Total diffusion volume	-54.6 ± 32.1*	45.7 ± 45.5	0.04^+^
Mean ADC	25.5 ± 32.7*	1.46 ± 7.9	0.04^+^
Skewness	-939 ± 842	-2.13 ± 69.8	0.29
Kurtosis	-27.5 ± 40.4	-1.56 ± 52.3	0.14
25th percentile	45.3 ± 50.4	18.0 ± 19.7	0.22
75th percentile	67.7 ± 61.8	55.7 ± 26.2	0.76
Spinal DWI			
Perfusion fraction	-26.4 ± 11.9	-21.0 ± 28.3	0.84
D*	220.6 ± 237.1	682.1 ± 635.5	0.35
mDixon Quant			
Fat fraction	94.3 ± 45.5*	24.7 ± 34.1	0.02^+^

Values represent mean ± standard deviation or standard error of percentage change, % except for kappa/lambda ratio.

*P < 0.05 compared with baseline within group.

^+^P < 0.05 change in CR/VGPR group compared with PR/SD/PD group.

CR, complete response; VGPR, very good partial response; PR, partial response; SD, stable disease; PD, progressive disease; BM, bone marrow; ADC, apparent diffusion coefficient; D*, pseudodiffusion coefficient.

The general linear model demonstrated that percentage changes in FF and M protein contributed significantly to CR/VGPR achievement (Table 4, P = 0.02, P = 0.04, respectively).

**Table 4:** General linear model examining the influence of clinical indices for predicting achievement of CR/VGPR.

Variable	*β ± standard error	Odds ratio (%95 CI)	*P*
Serum M protein (mg/dl)	-0.036 ± 0.018	0.96 (0.93–0.99)	0.04
Fat fraction (%)	0.082 ± 0.035	1.09 (1.01–1.16)	0.02

*β, partial regression coefficient.

**Fig 2:**
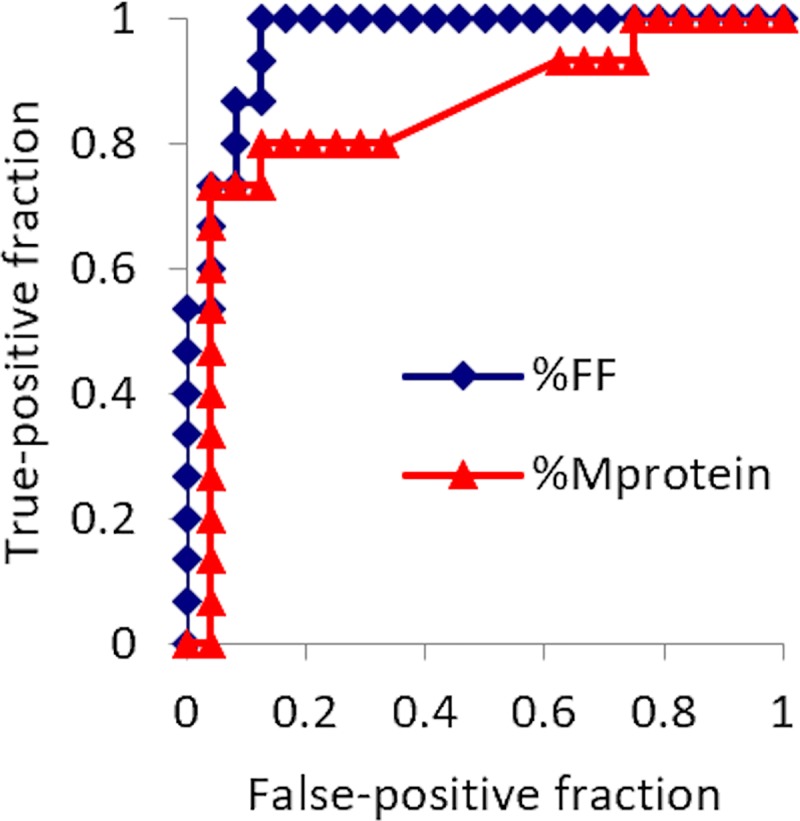
Receiver operating characteristics (ROC) curve for fat fraction (FF) of lumbar bone marrow by mDixon Quant sequence and serum M protein for predicting achievement of CR/VGPR. Areas under the ROC curve are 0.964 for FF and 0.847 for M protein.

Areas under the ROC curve were 0.964 for FF and 0.847 for M protein (Table 5 and Fig 2). Cutoff thresholds of a > 6.5% increase in FF allowed differentiation of patients who would achieve CR/VGPR with 93.3% sensitivity and 88.6% specificity. A cutoff threshold of a < 27.3% decrease in serum M protein allowed differentiation of patients who would achieve CR/VGPR with 80.0% sensitivity and 94.3% specificity.

**Table 5:** ROC results of parameters for predicting patients achieving CR/VGPR.

Variables	AUC ± standard error	Confidence interval	Sensitivity (%)	Specificity (%)	Cutoff
Serum M protein	0.847 ± 0.071	0.71, 0.99	80.0 (12/15)	94.3 (33/35)	< -27.3 (%)
Fat fraction	0.964 ± 0.025	0.91, 1.01	93.3 (14/15)	88.6 (31/35)	> 6.5 (%)

Data in parentheses represent numbers used to calculate percentages.

**Fig 3:**
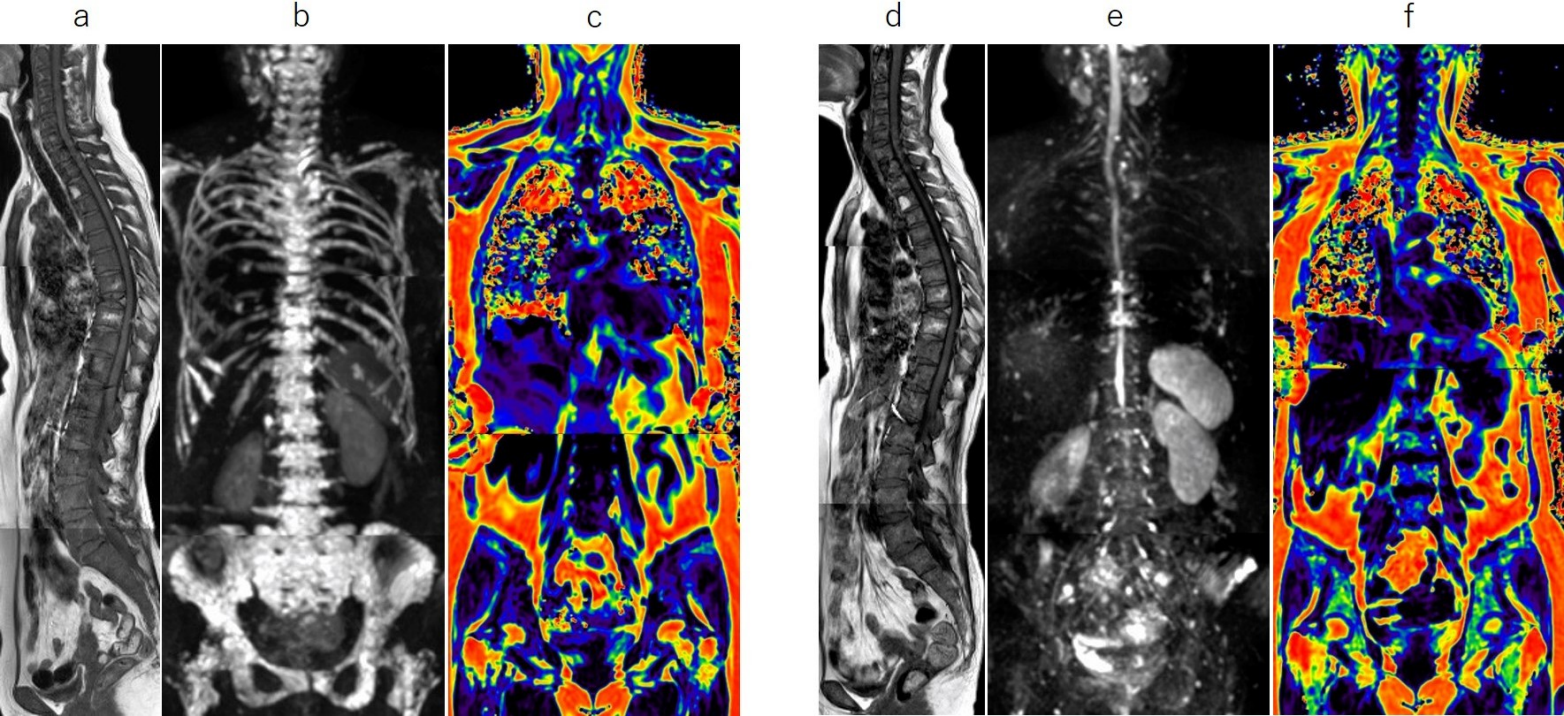
Whole-body MRI with a diffuse-dominant pattern. Sagittal T1-weighted images (T1WI, a), whole-body diffusion-weighted MRI (WB-DWI, b), and fat fraction (FF) map (c) at baseline, and corresponding T1WI (d), WB-DWI (e), and FF map (f) at 2 cycles of chemotherapy for a 60-year-old woman with symptomatic myeloma who relapsed after autologous stem cell transplantation. MRI shows diffuse low signal in the spine on T1WI (a) and diffuse high signal in the axial skeleton on WB-DWI (b) at baseline. After 2 cycles of chemotherapy with carfilzomib, lenalidomide, and dexamethasone, total diffusion volume (tDV) has significantly decreased (from 201 ml to 21 ml) and the FF (f) in the lumbar bone marrow has significantly increased (from 2% to 25%), indicating a decrease in the tumor mass.

**Fig 4:**
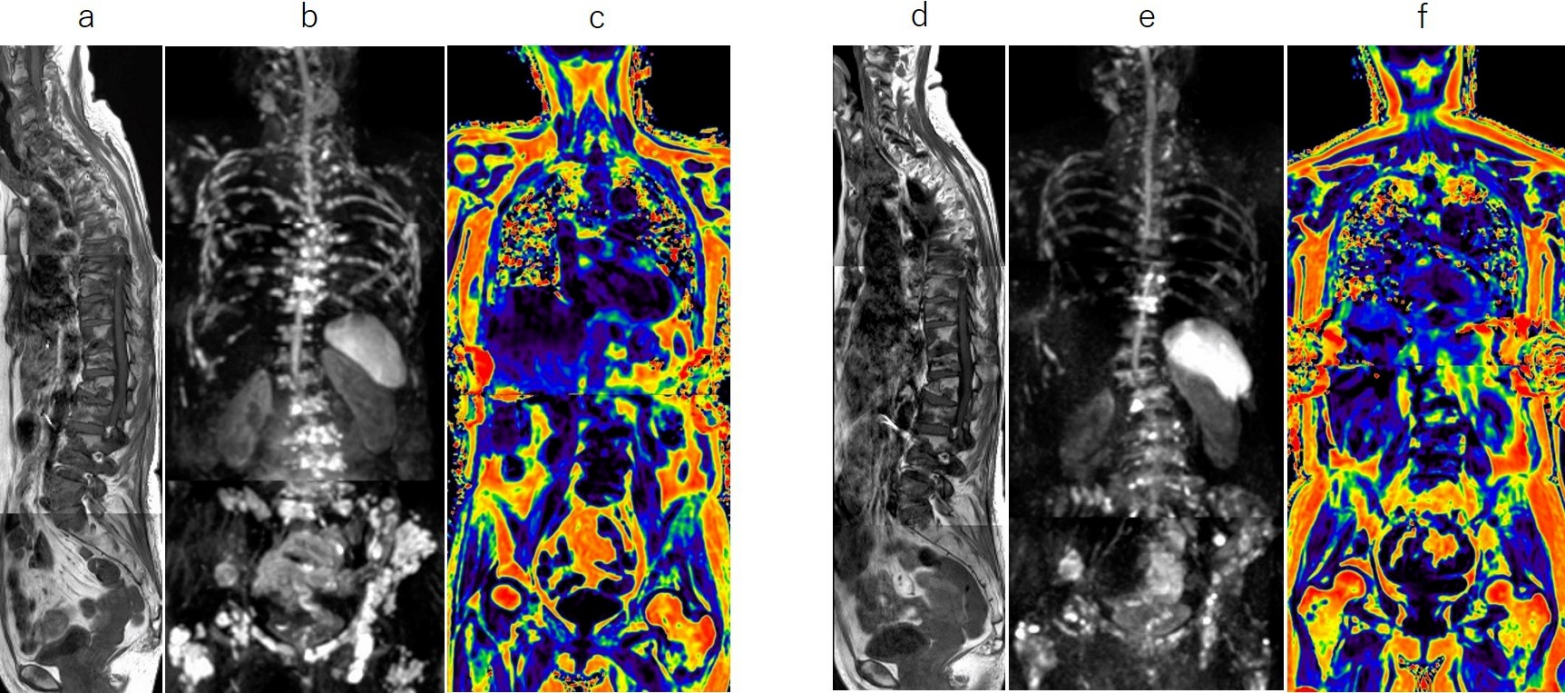
Whole-body MRI with a combined diffuse and focal pattern. Sagittal T1-weighted images (T1WI, a), whole-body diffusion-weighted MRI (WB-DWI, b), and fat fraction (FF) map (c) at baseline, and corresponding T1WI (d), WB-DWI (e), and FF map (f) at 2 cycles of chemotherapy for a 64-year-old woman with symptomatic myeloma who relapsed after chemotherapy with carfilzomib, lenalidomide, and dexamethasone. MRI shows focal bone lesions and heterogeneous low signal in the spinal bone marrow on T1WI (a). After 2 cycles of chemotherapy with pomalidomide and dexamethasone, total diffusion volume has decreased (from 263 ml to 176 ml), but FF in lumbar bone marrow has not shown significant change (from 15% to 16%).

**Fig 5:**
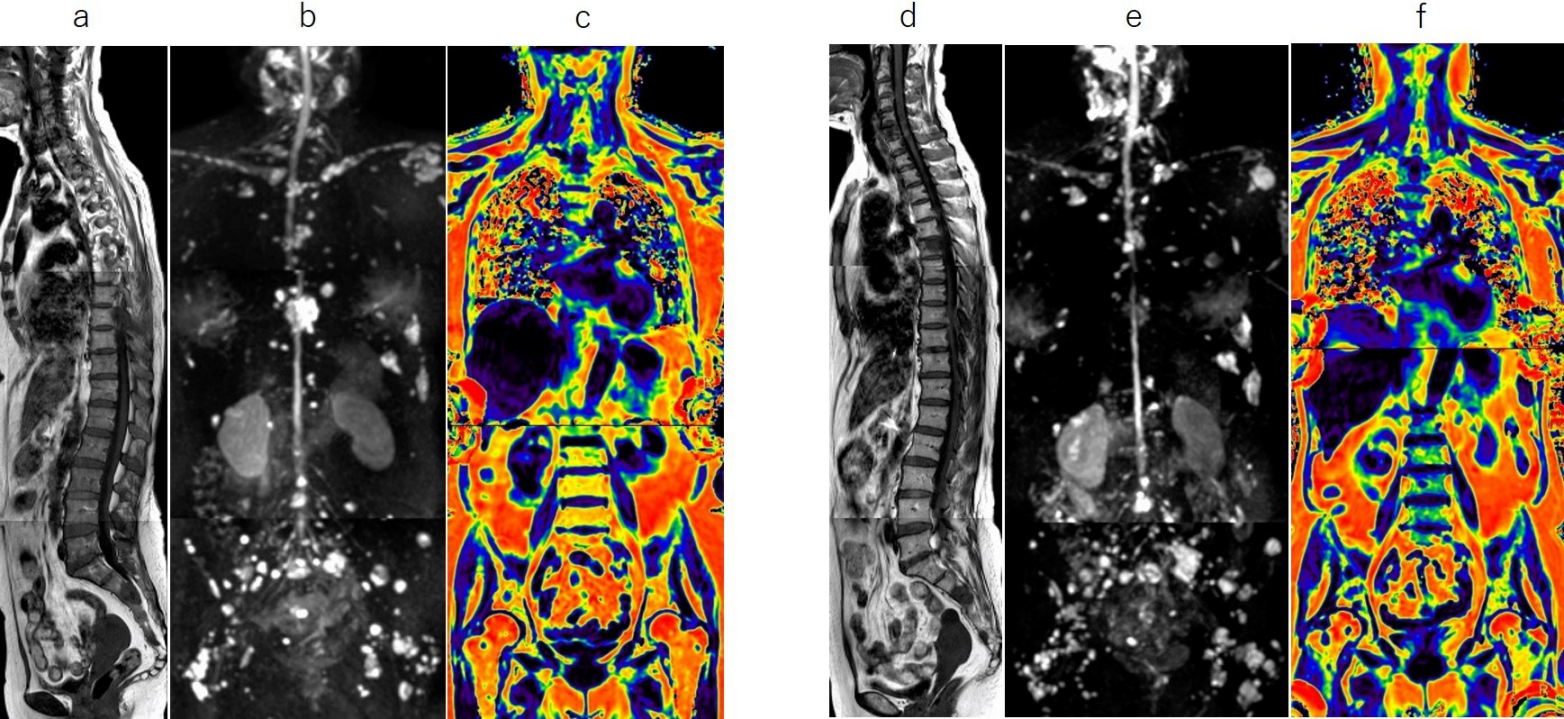
Whole-body MRI with a focal-dominant pattern. Sagittal T1-weighted images (T1WI, a), whole-body diffusion-weighted MRI (WB-DWI, b), and fat fraction (FF) map (c) at baseline, and corresponding T1WI (d), WB-DWI (e), and FF map (f) at 2 cycles of chemotherapy for a 70-year-old woman with symptomatic myeloma. MRI shows numerous focal bone lesions throughout the body at baseline. After 2 cycles of chemotherapy with bortezomib and dexamethasone, total diffusion volume does not show any significant change (from 47 ml to 49 ml), but FF in the lumbar bone marrow has decreased by 30% (from 56% to 40%), indicating an increase in diffuse infiltration into bone marrow by myeloma.

Representative images are shown in Figs 3–5.

## Discussion

We examined the comparative diagnostic performance of whole-body MRI and lumbar spinal MRI for predicting remission status in patients with MM. To the best of our knowledge, this article is the first to compare tDV calculated from WB-DWI and FF of lumbar BM measured soon after 2 cycles of chemotherapy in terms of the correlation of treatment response at completion of chemotherapy.

Whole-body MRI usually includes WB-DWI, which is based on changes in the Brownian motion of water molecules caused by tissue microstructure. In biological tissue, diffusion is restricted by interactions with cell membranes and macromolecules, and the degree of restriction correlates with microstructural factors such as tissue cellularity and the integrity of cell membranes [28, 29]. The ADC is a quantitative measure of Brownian movement and correlates inversely with tissue cellularity [30]. Use of the ADC also allows quantitative analysis of BM [31] and the ADC correlated with BM cellularity in patients with MM [32].

Several reports have examined the prognostic value of tDV for metastatic bone tumors. In 2016, metastatic bone tumor from castration-resistant prostate cancer was evaluated with tDV generated by WB-DWI [33]. The tDV showed correlations with established prognostic biomarkers, including prostate-specific antigen level and bone scan index, and was associated with overall survival for the disease. Blackledge et al. [34] evaluated response to treatment using tDV in patients with bone metastases from breast or prostate cancer. They demonstrated that non-responding patients showed a greater increase in tDV than responding patients. In the same year, responders to olaparib were reported to show a decrease in tDV, while no decrease was observed in any non-responders among patients with castration-resistant prostate cancer [35]. In those reports, abnormal signal areas that showed high signal intensity on DWI and low signal intensity on T1-weighted images were delineated as metastatic bone tumors and diffusely infiltrating tumors were not included in the study cohorts.

Evaluation of non-focal lesion (i.e., combined diffuse and diffuse-dominant pattern) is important for MM, because symptomatic myeloma patients with a diffuse pattern of marrow involvement at staging have been shown to experience worse prognosis [36–38]. In this study, tDV obtained by WB-DWI did not prove to be a significant predictor of CR/VGPR. We measured tDV as a surrogate marker for myeloma tumor mass using the reported mean ADC of the area corresponding to the area showing diffuse signal hypointensity on T1-weighted images based on visual inspection as the ADC threshold value [10] over which tissue was assumed to correspond to more advanced myeloma. Voxels in the BM with mild to moderate infiltration of myeloma cells therefore might not have been extracted and this might have led to underestimation of tumor volume, while the volume of focal lesions might have been more directly reflected by tDV. We determined the threshold according to a previous report [10] and subsequent preliminary experiment, simultaneously confirming maximal suppression of signal from normal/benign tissue without suppressing signal from diseased areas by visual inspection. By lowering the threshold, greater tumor burden was extracted, but residual signal from normal structures or artifacts might have been included. We therefore consider that the tDV used in this study was appropriate. Underestimation of tumor volume may also be due to the low spatial resolution of WB-DWI, in which small focal bone lesions are obscured by partial volume effects.

On the other hand, an early increase in the FF of lumbar BM soon after induction of chemotherapy correlated significantly with achievement of CR/VGPR. We consider that FF calculated from mDixon Quant, which utilizes signal cancellation of water- and fat protons within a voxel, might be more sensitive to even small amounts of tumor mass than tDV, which is estimated based on a certain threshold.

Absolute values of mean FF before chemotherapy tended to be lower for the CR/VGPR group (32.2 ± 24.7%) than for the PR/SD/PD group (44.2 ± 22.4%), but this finding did not reach the level of statistical significance (*P* = 0.14). Therefore, an early percentage change in FF is considered to offer a better predictor of achieving CR/VGPR than absolute mean FF at baseline examination.

One of the limitations of whole-body MRI for patients with MM is the prolonged acquisition time, which is typically 30–45 min. According to a previous report [39] with a mean acquisition time of 49 min, a substantial proportion of patients (86%) found the experience of whole-body MRI either not at all or not too unpleasant. However, half of the MRI examinations in our study were performed during chemotherapy, so the performance status of patients might have been worse than the status of patients in that previous report. Lumbar MRI and FF of lumbar BM could offer a surrogate for whole-body MRI or FDG-PET/CT to estimate treatment response during the course of chemotherapy for patients who have obvious lesions in the lumbar spine before treatment and cannot tolerate long scans, given the shorter acquisition time and reduced need for heavy receiver coils.

In this study, the sensitivity of FF from lumbar BM for identification of CR/VGPR was higher than that of serum M protein. This result may indicate that assessment of FF from lumbar BM may be useful for patients with Bence-Jones myeloma and non-secretory myeloma, in which M protein is not secreted into the blood.

In this study, one of the 15 patients in the CR/VGPR group was classified as false-negative based on the FF from lumbar BM. The pattern of BM infiltration in that patient was focal-dominant and the lumbar BM showed homogeneous high signal on T1-weighted images, suggesting the FF of the lumbar BM was within normal range. After 2 cycles of chemotherapy, focal lesions had decreased in size, although the FF showed no change. This may suggest that the change in myeloma tumor mass after chemotherapy is not reflected in lumbar BM for patients with a pure focal-dominant BM infiltration pattern. On the other hand, tDV of all patients in the CR/VGPR group decreased after 2 cycles of chemotherapy, at least to some extent.

Four of the 18 patients whose results were PD were classified as false-negative based on the FF of lumbar BM. In three of these four patients, the pattern of BM infiltration was focal-dominant. We attribute this to the focal-dominant BM infiltration pattern, as in the patient who achieved CR as described above. After 2 cycles of chemotherapy, the size of focal lesions and tDV in these patients did not significantly change. The other patient who had a combined diffuse and focal pattern BM infiltration pattern with decreased tDV was confirmed to have PD due to elevated sFLC and worsening neck pain.

In two of the 18 patients with PD, tDV did not show any significant change after 2 cycles of chemotherapy. These patients had a focal-dominant BM infiltration pattern and the FF of lumbar BM significantly decreased. This can be considered to reflect an increase in diffuse infiltration into BM by myeloma cells, supported by increases in sFLC and M protein in these patients.

One of the disadvantages of MRI is the relatively high frequency of false-positive results because of persistent nonviable lesions [40, 41]. In 2015, the consensus statement on the role of MRI in the management of patients with MM from the IMWG suggested that combined use with methods revealing active lesions (i.e., FDG-PET/CT) might be of greater value in evaluating the response to antimyeloma therapy [42]. In addition, FDG-PET/CT revealed faster changes to imaging findings than MRI in patients who responded to therapy [43].

The relationship between an early change after induction of chemotherapy in MRI and treatment outcome has not been fully described in previous works. To improve the results of MRI for more accurate prediction of remission status compared to FDG-PET/CT, Giles et al [9] used the mean ADC of the BM measured at induction and at a median of 13 weeks after beginning treatment. That study demonstrated that an increase in mean ADC by 3.3% was associated with response, having 90% sensitivity and 100% specificity. In their study, responders were defined as those showing CR, VGPR, or PR, and treatment outcome for 14 of the 21 patients was confirmed PR. Most studies on chemotherapy including autologous stem cell transplantation among newly diagnosed patients demonstrated that achieving CR or at least VGPR was associated with a longer progression-free survival and usually longer overall survival compared to patients who had PR or less [44–46]. Focusing on patients who achieved CR or VGPR is thus likely to be more related to actual clinical significance than defining a responder, which includes PR for predicting treatment outcome.

Several limitations to this study need to be considered when interpreting the results. First, this study was undertaken with a small sample size. Further prospective studies including integration of indices for deeper response such as stringent CR or molecular CR may be helpful to explore the potential role of MRI during chemotherapy. Furthermore, as mentioned, we used tDV calculated from WB-DWI as a surrogate marker for the myeloma tumor mass. To use ADC for the assessment of therapy response, assessments of measurement reproducibility are needed. According to previous reports, ADC in the tumor during chemotherapy changes within the range of 10–20% for hepatocellular carcinoma [47] and 8–25% for liver metastases from stomach and colorectal cancers [48]. In this study, the mean percentage change in ADC between the two MRI examinations was 25.5%. We therefore considered it unlikely that this limitation critically biased our results. Third, the patients did not have a standardized treatment protocol and some patients had a history of previous therapies. However, the purpose of this study was to compare the performance of imaging sequences for predicting chemotherapy outcome per patient. This limitation was therefore not considered critical.

In conclusion, an early increase in FF of lumbar BM and a decrease in serum M protein soon after induction of chemotherapy contributed significantly to the prediction of CR/VGPR status. Absolute value of FF at baseline did not provide a significant predictor of CR/VGPR. Results of this study may indicate that prediction of remission status can be achieved by assessing BM on lumbar spinal MRI with the mDixon Quant sequence. FF of lumbar BM could yield false-positive or false-negative results in patients with focal-dominant BM infiltration pattern because of the paucity of diffusely infiltrating myeloma cells. For all patients in the CR/VGPR group, tDV showed an early decrease, at least to some extent, but it did not prove to be a significant predictor of CR/VGPR.
